# A case of pregnancy with Rhesus antibody and bicornuate uterus - a favourable outcome: a case report

**DOI:** 10.1186/1757-1626-3-50

**Published:** 2010-02-03

**Authors:** Santanu Acharya, Samia Ahmed

**Affiliations:** 1 Department of Obstetrics & Gynaecology, Calderdale Royal Hospital, Salterhebble, Halifax, HX3 0PW, UK

## Abstract

**Introduction:**

In 1% of Rhesus negative women sensitisation occurs without any overt sensitising event during pregnancy. This accounts for late immunisation during a first pregnancy and is responsible for 18-27% of cases of alloimmunisation. The incidence of congenital uterine anomalies in a fertile population is 3.2% of which 5% are bicornuate uterus. Bicornuate uterus can lead to early miscarriages, preterm labor, fetal growth retardation and congenital malformations.

**Case presentation:**

A 23-year-old lady in her first pregnancy developed Anti-D antibodies at 28 weeks of gestation without any known sensitising event. In view of increasing anti-D titres, at 36 weeks she was delivered. Incidentally during caesarean section she was found to have bicornuate uterus. The neonate was treated with phototherapy and blood transfusion following delivery.

**Conclusion:**

Rhesus antibodies when managed by close monitoring and timely delivery can lead to favourable outcome. Bicornuate uterus does not always lead to complications like miscarriage, growth retardation or preterm labour and does not need any special intervention.

## Introduction

Human blood is classified according to two main systems: the ABO system and the Rhesus (Rh) system. The Rh system consists of several related proteins, the most important of which is called the Rhesus D (RhD) antigen. People who have this antigen on their red blood cells are said to be RhD positive, whereas those who do not are said to be RhD negative. A baby inherits its blood type from both parents. Therefore a mother who is RhD negative can carry a baby who is RhD positive. During pregnancy small amounts of fetal blood can enter the maternal circulation (an event called feto-maternal haemorrhage or FMH). The presence of fetal RhD-positive cells in her circulation can cause a mother who is RhD negative to mount an immune response, producing a template for the production of antibodies as well as small amounts of antibodies against the RhD antigen (anti-D antibodies). This process is called sensitisation or alloimmunisation. Sensitisation is unlikely to affect the current fetus but may result in haemolytic disease of the newborn (HDN) during a second RhD-positive pregnancy. These can be in situations in which FMH is likely (after delivery, miscarriage, abortion, invasive procedures or abdominal trauma) or even without any known sensitising events in 1% of the cases. In fact the most important cause of anti-D antibodies is now immunisation during pregnancy where there has been no overt sensitising event. Late immunisation during a first pregnancy is responsible for 18-27% of cases. In its mildest form the infant has sensitised red cells, which are detectable only in laboratory tests; however, HDN may result in jaundice, anaemia, developmental problems or intrauterine death.

Routine antenatal anti-D prophylaxis (RAADP) can be given to RhD-negative women to prevent sensitisation and hence prevent HDN. A recent health technology appraisal of RAADP forms the national guidance in the UK that RAADP be offered to all non-sensitised pregnant women who are RhD negative [1].

## Case presentation

A 23-year-old British Asian lady presented in her first pregnancy and had a dating scan in the first trimester. She was fit and healthy and did not have any significant medical or surgical history except mild asthma. All her booking bloods were normal. Her blood group was O Rhesus negative (C-c+E-e+K-). The antibody screen was negative and as per our hospital protocol she was due for another antibody check at 28 weeks' gestation. Her anatomy scan at 20 weeks was normal. Her pregnancy went on uneventfully.

At her 28 week visit she was given 1500 IU of Anti-D for prophylaxis. Blood taken for antibody screen surprisingly showed Allo Anti-D levels of 3 IU/ml. She did not have any history of trauma or blood transfusion which could have made her sensitised. She was told that anti-D levels of <4 IU/ml would be very unlikely to cause haemolytic disease of the newborn (HDN). However, she would from then on be under the care of a hospital obstetric unit with close follow-up. Guidelines recommended repeat testing every 2 weeks until delivery. Her husband's blood group was then checked and found to be B Rhesus positive (C+c-E-e+K-). The Rh phenotype indicated likely homozygous D expression, in which case all his children were likely to inherit the D antigen.

Two weeks later repeat testing showed significant increase in Anti-D levels at 8 IU/ml. Ultrasound scan for growth and liquor volume was normal. In another two weeks anti-D levels were 18 IU/ml. Levels above 15 IU/ml has a high risk of HDN. The HDN risk increases if gestation continued beyond term. The pregnancy was cautiously allowed to continue until 36 weeks with steroid prophylaxis to attain fetal lung maturity. USS performed showed normal growth and liquor volume. Umbilical and middle cerebral artery dopplers were also normal. After discussion with the patient induction of labour was started at 36^+3 ^weeks of gestation. She was given two doses of prostaglandin per vagina but with a modified Bishop's score of only 3 artificial rupture of membranes was not possible. She declined further attempt at induction of labour and underwent a Caesarean section (CS).

Just before CS fetal heart was found to be decelerating. During CS it was found that she had bicornuate uterus, which was not known beforehand, the pregnancy being located in one of the horns of the uterus. The CS was uneventful. A female baby weighing 2.6 Kg was born. Apgar scores were 5 @ 1 min, 7 @ 5 min and 9 @ 10 min. Blood gas analysis showed arterial P_H _7.07, Base excess (BE) - 5.9, venous PH 7.11, BE - 4.7. Neonatal resuscitation involved bag and mask ventilation and the baby was moved to Special Care Baby Unit. Cord blood Direct Antibody Test (DAT) was performed which was strongly positive. On day one following delivery hemoglobin and bilirubin were 14.1 gm/dL and 59 μmol/L respectively. The baby developed jaundice within the first 24 hours. She gradually developed increasing serum bilirubin levels and her haemoglobin also dropped. Treatment involved phototherapy and blood transfusion. However, she was discharged in good condition following two weeks' stay in the hospital when her anemia and jaundice were corrected.

## Discussion

Sensitisation can happen at any time during pregnancy, but is most common in the third trimester and during childbirth. Sensitisation can follow events in pregnancy known to be associated with FMH but can also occur in the absence of an observed potentially sensitising event. The risk of sensitisation is greatest in the first pregnancy and decreases with each subsequent pregnancy. Once sensitisation has occurred it is irreversible. In our case no known sensitising event was present to attribute for the development of the allo-antibodies at 28 weeks of gestation [1]. The diagnostic accuracy of non-invasive fetal Rh determination using maternal peripheral blood is 94.8%. Its use can be applicable to Rh prophylaxis and to the management of Rh alloimmunized pregnancies [2].

When red blood cells are broken down, bilirubin is released. In utero this is cleared by the placenta and is not harmful. However, after birth the neonatal liver cannot cope with the excess production of bilirubin, and this leads to jaundice (haemolytic disease of the newborn or HDN). Low levels of jaundice are not harmful but, if left untreated, higher levels can result in damage to specific areas of the neonatal brain, causing permanent brain damage (kernicterus). This can lead to a range of neurodevelopmental problems, such as cerebral palsy, deafness, and motor and speech delay. Postnatal jaundice can be treated with phototherapy and exchange transfusion. Our case was successfully managed by close monitoring, phototherapy and blood transfusion.

The risk of RhD alloimmunisation during or immediately after a first pregnancy is about 1.5%. Administration of 100 ug (500 IU) anti-D at 28 weeks and 34 weeks gestation to women in their first pregnancy can reduce this risk to about 0.2% without, to date, any adverse effects. Although such a policy is unlikely to confer benefit or improve outcome in the present pregnancy, fewer women will have Rhesus D antibodies in their next pregnancy. Adoption of such a policy will need to consider the costs of prophylaxis against the costs of care for women who become sensitised and their affected infants, and local adequacy of supply of anti-D gammaglobulin [3]. Potentially sensitising events introduce a quantity of fetal RhD antigen into the maternal circulation. The anti-D immunoglobulin administered neutralises this fetal antigen. In addition, anti-D immunoglobulin can be administered routinely in the third trimester as prophylaxis against small amounts of FMH that can occur in the absence of observable sensitising events. This is known as routine antenatal anti-D prophylaxis (RAADP). The use of anti-D immunoglobulin for RAADP is in addition to the administration of anti-D immunoglobulin following potentially sensitising events, and its use in either indication is not affected by prior use in the other [4].

**Figure 1:**
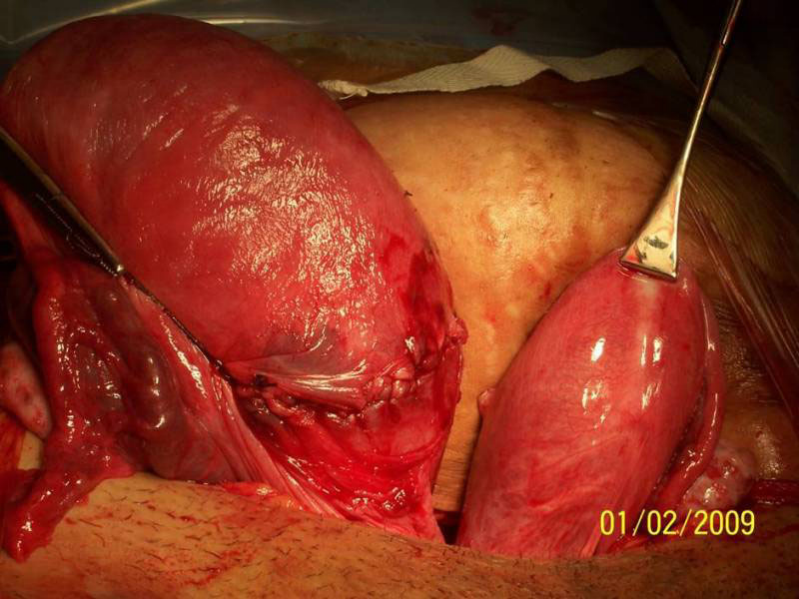
**Bicornuate uterus during Caesarean Section**. This shows the bicornuate uterus delivered outside the abdominal cavity during the operation.

This lady incidentally was found to have a bicornuate uterus during CS (Figure 1). The incidence of congenital uterine anomalies in a fertile population is 3.2%, 90% of which are septate uterus and another 5% each are bicornuate uterus and uterine didelphys [5]. A large case control study by the Spanish Collaborative Study of Congenital Malformations looked at 26945 consecutive malformed infants and assessed the frequency of congenital anomalies in offsprings of mothers with bicornuate uterus. The risk of congenital defects was found to be four times higher in mothers with bicornuate uterus in this study [6]. Another retrospective longitudinal study on uterine anomalies demonstrated live birth rates of 62.5% in cases of bicornuate uterus but early miscarriages and preterm labour were more common [7]. The cause for fetal distress in this case just before CS was not known. However, bicornuate uterus in this case had no untoward implication as far as this pregnancy was concerned.

This case demonstrates that a favourable outcome is possible in cases of Rhesus antibodies if proper monitoring is done. It also suggests that women with bicornuate uterus could have good reproductive prognosis without any intervention.

## Conclusion

Rhesus antibodies when managed by close monitoring and timely delivery can lead to favourable outcome. Bicornuate uterus does not always lead to complications like miscarriage, growth retardation or preterm labour and does not need any special intervention.

## Patient's perspective

My husband and myself was trying for a pregnancy for the previous eight months so was very excited when we found that I was pregnant. Everything was going on well, except the bad morning sickness that I had, until I was 28 weeks pregnant. At that point, the consultant mentioned to me that my blood group was O negative and I had rising anti-D levels which was a risk to my baby. After weeks of close monitoring I was finally told I was going to be induced at 36 weeks. I was very scared and nervous as I did not know what to expect. I was induced for two days with two pessaries but nothing happened. I requested a Caesarean section but the doctors and midwives persuaded me not to and carry on with the induction process. However, I was adamant and felt I cannot carry on further with the induction process. Finally the doctors agreed and I underwent the Caesarean section under a spinal anaesthetic with my husband being present in theatres. Dr Acharya performed my surgery and he was fantastic. At the end of surgery he told me that I had a double uterus. I was shocked and very surprised since nobody picked it up in any of my previous scans. I had never heard of anyone having a double uterus before! I am a little worried if it will affect any future pregnancies. My baby girl Lailah Noor was born and weighed 5.11 lbs. She was taken straight to SCBU as she was severely anaemic and became jaundiced. After two units of blood transfusion baby Lailah is doing well and gaining weight at present.

## Consent

Written informed consent was obtained from the patient for publication of this case report and accompanying images. A copy of the written consent is available for review by the Editor-in-Chief of this journal.

## Competing interests

The authors declare that they have no competing interests.

## Authors' contributions

SAA was involved in the treatment, performed the surgery and prepared the manuscript. SAD was the patient who wrote the patient's perspective. Both authors read and approved the final manuscript.
